# Investigation into the Neuroprotective and Therapeutic Potential of Plant-Derived Chk2 Inhibitors

**DOI:** 10.3390/ijms25147725

**Published:** 2024-07-15

**Authors:** Monika Kisielewska, Michał Filipski, Kamil Sebastianka, Dobrawa Karaś, Klaudia Molik, Anna Choromańska

**Affiliations:** 1 Faculty of Medicine, Wroclaw Medical University, Mikulicza-Radeckiego 5, 50-345 Wroclaw, Poland; monika.kisielewska@student.umw.edu.pl (M.K.); michal.filipski@student.umw.edu.pl (M.F.); kamil.sebastianka@student.umw.edu.pl (K.S.); dobrawa.karas@student.umw.edu.pl (D.K.); klaudia.molik@student.umw.edu.pl (K.M.); 2 Department of Molecular and Cellular Biology, Faculty of Pharmacy, Wroclaw Medical University, Borowska 211A, 50-556 Wroclaw, Poland

**Keywords:** plant-derived inhibitors, innovative drugs, cancer treatment, checkpoint kinase 2, DNA repair, apoptosis, cell cycle arrest, checkpoint kinase 2 inhibitors, neurodegenerative diseases, amyotrophic lateral sclerosis

## Abstract

Nature provides us with a rich source of compounds with a wide range of applications, including the creation of innovative drugs. Despite advancements in chemically synthesized therapeutics, natural compounds are increasingly significant, especially in cancer treatment, a leading cause of death globally. One promising approach involves the use of natural inhibitors of checkpoint kinase 2 (Chk2), a critical regulator of DNA repair, cell cycle arrest, and apoptosis. Chk2’s activation in response to DNA damage can lead to apoptosis or DNA repair, influencing glycolysis and mitochondrial function. In cancer therapy, inhibiting Chk2 can disrupt DNA repair and cell cycle progression, promoting cancer cell death and enhancing the efficacy of radiotherapy and chemotherapy. Additionally, Chk2 inhibitors can safeguard non-cancerous cells during these treatments by inhibiting p53-dependent apoptosis. Beyond oncology, Chk2 inhibition shows potential in treating hepatitis C virus (HCV) infections, as the virus relies on Chk2 for RNA replication in neurodegenerative diseases like amyotrophic lateral sclerosis (ALS), in which DNA damage plays a crucial role. Plant-derived Chk2 inhibitors, such as artemetin, rhamnetin, and curcumin, offer a promising future for treating various diseases with potentially milder side effects and broader metabolic impacts compared to conventional therapies. The review aims to underscore the immense potential of natural Chk2 inhibitors in various therapeutic contexts, particularly in oncology and the treatment of other diseases involving DNA damage and repair mechanisms. These natural Chk2 inhibitors hold significant promise for revolutionizing the landscape of cancer treatment and other diseases. Further research into these compounds could lead to the development of innovative therapies that offer hope for the future with fewer side effects and enhanced efficacy.

## 1. Introduction and Background

Nature is an infinite and unfailing source of compounds that serve many diverse purposes, the development of innovative drugs being one of them. Even though the constantly improving chemically synthesized therapeutics remain one of the major constituents in treating many diseases, natural compounds have started to assert themselves more and more [1]. As cancer remains one of the leading causes of death each year [2], researchers continue in their endeavors to discover new cures for the disease. One of the promising treatments involves utilizing natural inhibitors of checkpoint kinase Chk2 [3].

The cell cycle checkpoint kinase Chk2 is crucial in regulating DNA repair, cell cycle arrest, and apoptosis [3]. After receiving a signal indicating DNA damage, it can activate pathways resulting in either apoptosis or repair of the DNA [4]. The Chk2 kinase is a serine/threonine kinase responsible for phosphorylating more than 20 proteins when activated [5]. Chk2 controls glycolysis, the functioning of mitochondria, and DNA damage responses. For instance, it is known that cells with DNA damage and increased levels of Chk2 depend upon glycolysis for ATP synthesis, as their mitochondria become dysfunctional [6]. As one of the cell cycle checkpoint kinases, Chk2 can halt the cell cycle at G1/S and G2/M so that the developing cell does not progress further with structural abnormalities. Eventually, the cell can undergo one of a few processes, such as apoptosis, DNA repair, and senescence. Cancer growth and progression are often associated with malfunctioning cell cycle verification. Treatment with natural Chk2 inhibitors aims to inactivate DNA damage response pathways, including DNA repair and suspension of the cell cycle.

Consequently, this leads to the discontinuation of cancer development and eventually results in the death of cancer cells. Furthermore, natural Chk2 inhibitors can activate apoptosis, senescence, or mitotic catastrophe [7]. Some human tumor cells exhibit an increased amount of activated Chk2, implying that these cells may have conformed to the unceasing activation of Chk2 and have become somewhat addicted to its presence. Therefore, the inhibition of Chk2 in these cells may result in their death [4].

On the other hand, Chk2 inhibitors serve as protective agents for non-cancerous cells during cancer treatment, such as radiotherapy or chemotherapy [3,7]. This protection is attributed to Chk2’s role in activating p53-dependent apoptosis. Inhibiting Chk2 and deactivating the p53-dependent apoptosis pathway in non-cancerous cells ensures their survival during cancer treatment. Furthermore, Chk2 inhibitors are known to amplify the effects of radiotherapy and/or chemotherapy [1,4]. Blocking the DNA repair pathways in cancer cells through Chk2 inhibition can induce cell death and halt cancer progression.

Chk2 inhibition treatment shows significant potential in treating HCV infections, a critical area of research [8]. HCV, a single-stranded RNA virus, is the causative agent of hepatitis C [9]. The disease can progress to hepatocellular carcinoma and cirrhosis, and no vaccine is currently available. While antiviral medications like sofosbuvir and daclatasvir can cure the disease, Chk2 inhibition presents a promising alternative yet to be approved [4,10,11].

During hepatitis C infection, the virus causes cleavages in the host cell’s double-stranded DNA (dsDNA). A double-stranded DNA break (DSB) is one of the most detrimental types of DNA impairment. DSBs may cause further damage to the genome, including deletions, translocations, and amplifications. In the event of HCV infection, the virus increases the incidence of cellular gene mutations. HCV was proven to require Chk2 activity for its RNA replication [8,12]. The virus hijacks the molecular processes that enable the cell to express proteins through a consensual entry. The virus causes the mechanisms to produce proteins for the virus [13]. Thus, by implementing Chk2 inhibitors, it is possible to stop the spread of the virus and cure the patient.

Chk2 inhibition could also be used in the treatment of neurodegenerative diseases. The development of these diseases, such as Parkinson’s disease (PD), Alzheimer’s disease (AD), and amyotrophic lateral sclerosis (ALS), has been associated with DNA damage and an inadequate, continuous DNA damage response, as the cells are not able to keep up with retrieving the impaired DNA. DNA damage can occur during replication or transcription and due to the presence of reactive oxygen species (ROS) and genotoxic agents. DNA double-stranded breaks are hazardous in cells without the ability to replicate as they cannot be replaced. Hence, if there is an impairment in the DNA repair mechanisms, neuropathy may develop, leading to senescence and apoptosis. In neurons, Chk2 was discovered to act as a master regulator at the G1/S checkpoint, either allowing or ending the cell’s development, depending on the state of its DNA. Hence, Chk2 inhibition with plant-derived agents is believed to be an optimal target for establishing a new treatment for neuropathies. Furthermore, this type of treatment is also viable in patients with optic nerve or spinal cord injuries due to its neuroprotective properties [14].

Plant-derived inhibitors of Chk2 offer a promising future for treating many diseases. These therapeutics may provide treatment with milder side effects compared to conventional ones, especially chemotherapy or radiotherapy. Moreover, natural compounds can influence multiple metabolic pathways, thus increasing their efficacy. This review explores the potential of natural Chk2 inhibitors in various medical applications, particularly in oncology, infectious diseases, and neurodegenerative disorders. It will cover the mechanisms of Chk2 action, the role of Chk2 inhibitors in cancer treatment, their protective effects on non-cancerous cells during cancer therapy, and their therapeutic potential in treating HCV infections and neurodegenerative diseases. The review will also highlight specific plant-derived agents, such as artemetin, rhamnetin, pachypodolol, rhamnazin, rutin, curcumin, resveratrol, xanthatin, and berberine, and their roles in these contexts.

## 2. Chk2 Inhibition and Mechanisms

**Figure 1:**
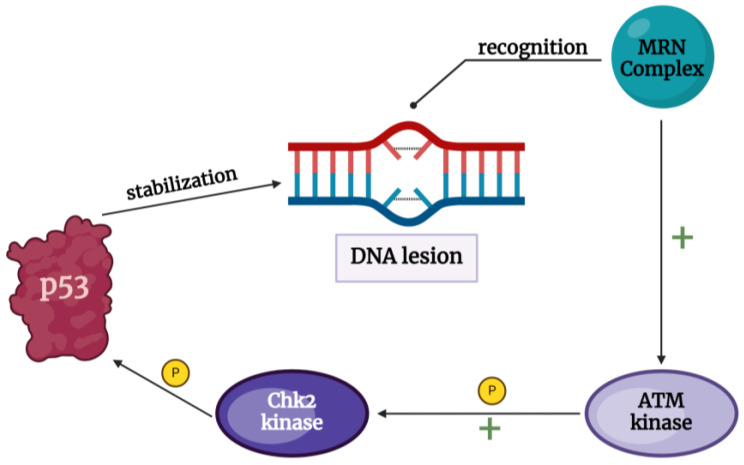
Response of Chk2 to DNA lesion [4,5,6,7].

Chk2 is a serine/threonine kinase that plays a vital role in cell cycle control and the regulation of responses to DNA lesions. The activity of Chk2 provides cellular integrity and an appropriate reaction to DNA damage, promoting its repair or apoptosis, depending on the cells’ genetic background and the damage’s gravity. Persistent double-strand breaks (DBSs), caused by endogenous and exogenous agents, are the main genotoxic factors that, when left unrepaired, lead to genome instability and, eventually, cell death. DNA lesions are recognized by the MRN complex (MRE11-RAD50-NBS1) that recruits ATM (ataxia telangiectasia-muted) kinase, which activates checkpoint kinase 2 (Chk2) through its phosphorylation. Activated Chk2 directly phosphorylates the p53 protein, promoting its stabilization and the regulation of its cellular response to DNA damage [4,7] (Figure 1). Thus, the ATM-Chk2 pathway is crucial in DNA damage response (DDR), inducing cell cycle arrest at the G1/S checkpoint and initiating repair or apoptosis.

In the case of neuron cells, DBSs are very dangerous because of the non-replicative character of neurons, which are terminally differentiated and cannot be rapidly substituted. Nonrepaired DBSs lead to the accumulation and invincible activation of DDR, which dysregulates the cell cycle [15]. As a result, DDR causes senescence and dysfunction in neurons, which trigger multiple acute and chronic pathological neurological conditions such as stroke, spinal cord injury, Alzheimer’s disease, Huntington’s disease, and Parkinson’s disease [16]. Because of its crucial role in the response to DNA damage, Chk2 has become an attractive therapeutic target that can prevent neurodegeneration and provide neuron protection—in the study conducted on *Drosophila*, a suppression of Chk2 kinase resulted in an apparent protective effect in neurons, where DBS occurred. In addition, knockdown of Chk2 in an in vitro model of a spinal cord injury significantly improved rat dorsal root ganglion neuron survival and stimulated its outgrowth and axon regeneration. Thus, Chk2 inhibition prevents neuronal decline, leading to neurodegeneration, and enhances axon regeneration and the restoration of lost functions after acute nervous system traumas [14].

The inhibition of Chk2 kinase is not just a theoretical concept, but a tangible advancement in cancer treatment. It has been reported to significantly boost the efficiency of therapeutic agents by sensitizing neoplastic cells to cytotoxic activity while desensitizing normal cells from DNA-damaging factors. Chk2 inhibitors have been shown to enhance the chemotherapeutic efficiency of the cisplatin-induced apoptosis of ovarian cancer cells, especially those with defective p53 [17]. Additionally, Chk2 inhibition raises the level of mitotic catastrophe and makes proliferating cells more sensitive to apoptosis induced by doxorubicin treatment [18]. This progress in cancer treatment, thanks to Chk2 inhibitors, is a testament to the relentless efforts of the scientific community to improve patient outcomes.

The molecular process of direct Chk2 inhibition consists of suppressing the activating phosphorylation reactions. The critical responses to Chk2 activation are its phosphorylation at Thr68 by ATM kinase, which leads to dimerization, trans-autophosphorylations at Thr383 and Thr387, and *cis*-phosphorylation at Ser516 [4].

A small group of bioactive molecules that occur in plants provides inhibitive abilities to Chk2 kinase and can be used in therapy (Table 1). The bioactive flavonoids discovered in *Miliusa,* such as artemetin, rhamnetin, pachypodolol, rhamnazin, and rutin, exhibit inhibitive abilities to Chk2 by interlinkage with catalytic amino acid residues. Molecular docking analysis demonstrated that rutin forms the strongest hydrogen bond interactions while attempting to use the least amount of binding energy. The overrepresented surrounding residue that formed a hydrogen bond with Chk2 in the case of rhamnazin, rhamnetin, and pachypodolol was Val313. Hydrophobic interactions are frequently carried out by Val415, Ile419, and Lys349 [19]. Naturally occurring polyphenols may also exhibit inhibitive properties by regulating the Chk2 gene expression. A study on Hep-2 cells showed that pretreatment with curcumin decreases the expression of ATM and Chk2 mRNA [20]. Another polyphenol, resveratrol, can reduce the phosphorylation of ATM and Chk2, caused by oxidative stress, by 60%. Pretreatment with resveratrol lowered the levels of phosphorylated histone H2AX (γH2AX), a marker of DNA breaks [21]. Finally, xanthatin, a sesquiterpene lactone found in the leaves of the *Xanthium strumarium* plant, downregulates the expression of Chk1 and Chk2 proteins, which are crucial for regulating the G2 checkpoint in response to DNA damage [22].

## 3. Neuroprotective Effects

The formation of DNA double-strand breaks (DSBs) is a frequently encountered form of DNA damage. While DNA damage in actively dividing cells leads to cell cycle arrest and facilitates repair mechanisms during cell cycle phases, post-mitotic neurons rely on a complex pathway called the DNA damage response (DDR). The DDR system determines whether neurons survive or undergo apoptosis. Unrepaired DSBs lead to prolonged activation of DDR, which may cause neurons to improperly re-enter the G1 cell cycle phase, resulting in neural dysfunction, apoptosis, and senescence. Senescent-like neurons are characterized by metabolic dysregulation, mitochondrial dysfunction, and the overproduction of pro-oxidant, pro-inflammatory, and matrix-remodeling factors, which can negatively affect neighboring cells and result in age-related conditions. This phenomenon may contribute to Alzheimer’s disease (AD), amyotrophic lateral sclerosis (ALS), cerebral ischemia, and stroke [14,15,23,24]. It has also been suggested that the persistent DDR and the inappropriate activation of the ATM signaling pathway may play a role in the development of fragile X syndrome and Huntington’s disease. It is becoming increasingly evident that DDR plays a significant role in neurodegenerative disorders [25].

Inhibiting the DDR has been shown to provide neuroprotection. Upon DNA damage, the initial response involves the MRN complex, comprising three proteins: meiotic recombination 11 (Mer11), radiation 50 (Rad50), and Nijmegen breakage syndrome 1 (NBS1). This complex binds to DSBs, activating the ataxia telangiectasia mutated (ATM) kinase. ATM then phosphorylates and activates checkpoint kinase 2 (Chk2), activating p53, which promotes apoptosis. Studies have demonstrated that, as previously mentioned, inhibitors targeting Mre11 and ATM can mitigate neurodegeneration in Drosophila models of Alzheimer’s disease and enhance axon regeneration after spinal cord injury (SCI) [15].

Recent research has found that inhibiting Chk2 also results in neuroprotection, axon regeneration, and a significant restoration of lost function following central nervous system (CNS) injury. Furthermore, Chk2 has fewer potential targets than ATM, suggesting it may be a more promising therapeutic target. The evidence indicates that relevant functional recovery can be achieved with only 30% inhibition of Chk2, indicating that low doses of Chk2 inhibitors, such as prexasertib, may be effective [14]. Other studies have further supported the above association between DDR and AD pathogenesis. It has been shown that elevated levels of Chk2 function as tau kinases. Tau, a protein responsible for regulating microtubule stability, can undergo abnormal phosphorylation, leading to the formation of tau aggregates known as neurofibrillary tangles (NFTs). This process is observed in multiple neurodegenerative disorders, including AD.

Overexpression of Chk2 has been demonstrated to phosphorylate tau at Ser262, consequently exacerbating tau toxicity in a transgenic Drosophila model [26,27]. Further findings indicate that suppressing the ATM/Chk2 pathway via wild-type p53-induced phosphatase 1 (Wip1) improves ALS. Wip1 dephosphorylates ATM, Chk2, and p53. It has been observed that increased Wip1 expression in the CNS delays the onset of disease symptoms, extends survival, reduces motor neuron loss, and enhances motor function in mice. Conversely, inhibiting Wip1 worsens disease progression [25].

Moreover, embryonic Wip1 deficiency results in a notable decrease in neural cells in the olfactory bulb, abnormal morphology of hippocampal neurons, impaired memory, and heightened anxiety- and depression-like behaviors in adult mice [28]. Another neurological disorder presenting the importance of the ATM/Chk2 pathway in its pathology is Huntington’s disease (HD). Persistent elevation of ATM signaling has been observed in cells obtained from mice with HD and in the brain tissue of both HD mice and patients. Treatment with ATM inhibitors prevented the death of rat striatal neurons induced by mutant Huntingtin (mHTT) and improved pluripotent stem cells derived from HD patients. Decreasing ATM signaling has been proposed as a potential strategy to alleviate mHTT toxicity in cellular and animal models of HD, underscoring ATM as a promising therapeutic target for HD [29].

Although still under investigation, using plant-derived Chk2 inhibitors presents a promising approach for preventing and treating neurological disorders. One of the plant-derived Chk2 inhibitors is resveratrol (RSV), a natural polyphenol found in grapes, peanuts, and soy. Studies indicate that RSV and red wine extract (RWE) prevent damage by inhibiting the ATM/Chk2 or Chk1 pathways. Pretreatment with RWE and RSV precluded the phosphorylation of ATM and Chk2 while preventing oxidative stress and protecting retinal pigment epithelial (RPE) cells against DNA damage by downregulating the DDR pathway [21]. Resveratrol is widely examined for its neuroprotective effects in both Parkinson’s disease (PD) and Alzheimer’s disease. Its mechanisms primarily involve reducing oxidative stress by scavenging reactive oxygen species (ROS) and preventing LDL peroxidation.

Additionally, it upregulates heme oxygenase 1 (HO-1), promotes autophagy, modulates mitochondrial dynamics, suppresses neuroinflammation, and activates sirtuin-1 (SIRT-1), further contributing to neuronal protection and memory improvement. RSV also inhibits tauopathy and amyloid beta (Aβ)-plaque formation, ultimately improving memory and preventing neuronal death in AD. Furthermore, resveratrol exhibits promising neuroprotective effects in experimental PD models induced by neurotoxins. In vitro and in vivo studies have demonstrated resveratrol’s ability to protect dopaminergic neurons and prevent motor and cognitive impairments caused by these neurotoxins. Moreover, it reduces α-synuclein-induced cell death and autophagic dysfunction. Combining resveratrol with conventional PD treatments like levodopa (L-DOPA) may offer synergistic benefits, indicating a promising avenue for future research in neurodegenerative diseases. However, these outcomes have not been successfully replicated in human studies, likely due to resveratrol’s poor bioavailability and other pharmacokinetic factors.

Further research and clinical trials are necessary to fully comprehend resveratrol’s therapeutic potential and safety in treating neurodegenerative diseases [30,31]. Notably, resveratrol has been shown to protect against brain damage resulting from cerebral ischemia and subsequent reperfusion. This protection may be achieved due to the activation of two signaling pathways: Janus kinase 2/signal transducer and activator of transcription 3 (JAK2/STAT3) and phosphoinositide 3-kinase/protein kinase B/mammalian target of rapamycin (PI3K/AKT/mTOR) [32].

Berberine (BBR), an alkaloid derived from Rhizoma coptidis, has been observed to have potential in the treatment of age-related diseases. It has been demonstrated that low doses of BBR can mitigate senescence in human diploid fibroblasts (HDFs). This protective effect involves reducing the loss of mitochondrial membrane potential and the production of intracellular reactive oxygen species (ROS), making cells less susceptible to hydrogen peroxide-induced cell cycle arrest and growth inhibition. Moreover, low BBR concentrations could modestly elevate ROS levels and enhance the expression of SIRT1. BBR has been shown to attenuate the activation of checkpoint kinase 2 (Chk2) induced by hydrogen peroxide, making it another potential plant-derived Chk2 inhibitor [33]. Berberine is a promising agent acting against neurological conditions such as AD, PD, HD, cerebral ischemia, depression, schizophrenia, and anxiety. Its neuroprotective effects are caused by engaging multiple molecular pathways, including reducing oxidative and endoplasmic reticulum stress, diminishing neuroinflammatory responses, inhibiting apoptotic pathways, promoting autophagy, adjusting neurotransmitter levels, and regulating cytochrome P450 enzyme activities and gut microbiota composition. These actions ultimately lead to a reduction in neuronal damage and cell death.

Furthermore, berberine effectively crosses the blood–brain barrier (BBB), allowing for the treatment of neurotoxic injuries induced by various factors such as drugs, environmental toxins, aging, and ischemia–reperfusion. Despite its potential, further research is necessary to fully understand its long-term effects and potential adverse reactions and elucidate the precise mechanisms underlying its therapeutic effects in neurodegenerative diseases for optimal clinical use. It is noteworthy that while oral administration of berberine is generally safe, injections may cause toxicity [34,35].

Another promising natural compound that could serve as a selective Chk2 inhibitor that potentially offers therapeutic benefits by modulating the DNA damage response in neural cells is eupatilin [36]. Eupatilin, a naturally occurring flavonoid extracted from Artemisia species, has garnered significant interest in recent years due to its diverse pharmacological properties, including anti-inflammatory, antioxidant, and anticancer activities. Of particular interest is its role as a selective checkpoint kinase 2 (Chk2) inhibitor and its potential neuroprotective effects, as demonstrated in preclinical studies [37]. One study showed that eupatilin significantly reduced neuronal apoptosis and oxidative stress in a model of Parkinson’s disease by inhibiting the Chk2 pathway [38]. Another study reported that eupatilin improved cognitive function and reduced neuroinflammation in a mouse model of Alzheimer’s disease, again implicating Chk2 inhibition as a critical mechanism [39]. Eupatilin’s antioxidant and anti-inflammatory properties further contribute to its neuroprotective effects. It has been shown to scavenge free radicals and upregulate antioxidant enzymes, thereby reducing oxidative damage in neural tissues.

Additionally, eupatilin attenuates neuroinflammation by inhibiting the activation of microglia and astrocytes, which are involved in the inflammatory response in neurodegenerative diseases [40]. Eupatilin presents a multifaceted approach to neuroprotection. Furthermore, its ability to cross the blood–brain barrier enhances its potential as a therapeutic agent for central nervous system disorders [41].

## 4. Therapeutic Potential and Future Directions

In recent years, herbal medicine has gained significant interest in scientific circles. Numerous scientific studies focus on natural substances. This is often due to the described substances’ relative safety and minimal side effects [42]. The aforementioned plant-derived Chk2 inhibitors may also be included in this group. They often exhibit comprehensive functions. For instance, rutin exhibits actions including anticarcinogenic, neuroprotection, antiproliferative, anti-inflammatory, and antimetastatic actions, and acts as an antioxidant via inhibiting lipid peroxidation and ameliorating oxidative stress. This diversity of positive effects makes it an attractive substance from a medical point of view [43].

Chk2 inhibitors hold significant therapeutic potential beyond neuroprotection, particularly in cancer therapy. Chk2 is a crucial cell cycle checkpoint and regulator of DNA damage response pathways. It plays a vital role in maintaining genomic integrity. Dysregulation of Chk2 has been implicated in various human cancers, making it an attractive target for therapeutic intervention [6,44]. By inhibiting Chk2 activity, Chk2 inhibitors can sensitize tumor cells to DNA-damaging agents, induce cell cycle arrest and apoptosis, and enhance the efficacy of conventional cancer treatments, such as chemotherapy and radiation therapy [45]. Additionally, Chk2 inhibitors may overcome drug resistance mechanisms, offering new strategies for combating cancer progression and improving patient outcomes [46]. In addition, another exciting property of Chk2 inhibitors, that was recently detected in in vitro *studies* is essential. Namely, healthy, non-tumor cells exhibit chemoprotective and radioprotective effects, offering prospects for reducing conventional cancer treatment’s toxicity and side effects [17,47].

Artemetin, a promising compound, has been shown to decrease cancer cell growth, induce apoptosis, and inhibit cell migration in various cancer cells, including neuroblastoma, hepatocarcinoma, and breast cancer cells. Studies have also identified that artemetin affects microtubule dynamics and modulates proteins like filamins, which are crucial for cell adhesion and migration. This modulation leads to cytoskeleton disassembly and inhibits cell migration, making artemetin a potential candidate for anticancer therapy by disrupting fundamental cellular processes involved in cancer progression [48].

Pachypodol, a bioactive flavonoid found in Pogostemon *cablin* (patchouli), is a fascinating compound with a wide range of biological activities. It has been identified as having anti-inflammatory, antioxidant, anti-mutagenic, antimicrobial, antidepressant, anticancer, antiemetic, antiviral, and cytotoxic properties. The pharmacological potential of pachypodol is highlighted by its ability to scavenge free radicals, inhibit mutagenic activity, and exhibit therapeutic effects in various disease models [49]. Additionally, pachypodol has been shown to attenuate cisplatin-induced liver dysfunction by inhibiting oxidative stress, inflammation, and apoptosis, indicating its potential as a protective agent in chemotherapy-related liver toxicity. Its potential applications in modern therapeutics underscore the need for further research to fully understand its mechanisms of action and clinical benefits [50].

Rhamnazin, a flavonoid derived from natural sources, has garnered attention for its potential as an inhibitor of checkpoint kinase 2 [19]. Moreover, it has been shown to inhibit angiogenesis, the new blood vessel formation process, which is crucial for tumor growth and metastasis. It exerts this effect primarily by targeting the VEGFR2 (Vascular Endothelial Growth Factor Receptor 2) signaling pathways. By inhibiting VEGFR2, rhamnazin prevents the phosphorylation of downstream targets such as MAPK, AKT, and STAT3, which are essential for endothelial cell proliferation, migration, and new blood vessel formation [51].

Rhamnetin enhances the sensitivity of non-small cell lung cancer (NSCLC) cells to radiation by suppressing Notch-1 expression. It inhibits epithelial–mesenchymal transition (EMT), which is crucial for cancer metastasis. By increasing the expression of tumor-suppressive microRNA, miR-34a, rhamnetin reduces Notch-1 expression and promotes apoptosis. In a mouse model, rhamnetin and irradiation significantly reduced tumor volume compared to irradiation alone [52]. Rhamnetin also increases the susceptibility of hepatocellular carcinoma (HCC) cells to chemotherapy and small molecular kinase inhibitors, making treatments more effective. By inhibiting the Notch-1 signaling pathway, which is involved in cell survival and drug resistance, rhamnetin reduces the expression of MDR-related proteins. It also enhances the expression of miR-34a, which helps downregulate pro-survival genes and MDR-related proteins. This suggests that rhamnetin could be a novel approach to overcoming multi-drug resistance in HCC therapy [53]. In addition to its effects on cancer cells, rhamnetin suppresses the production of pro-inflammatory cytokines and mediators in mouse macrophages by acting on the p38 MAPK, ERK, JNK, and COX-2 pathways. This anti-inflammatory action further highlights its potential therapeutic benefits [54].

Furthermore, rhamnetin exhibits strong antioxidant properties, protecting cells from oxidative stress by enhancing the expression of catalase and Mn-SOD and inhibiting the production of intracellular reactive oxygen species (ROS). It protects cardiomyoblast cells from H_2_O_2_-induced apoptosis, improving cell viability and reducing cell death. By activating signaling pathways such as Akt/GSK-3β and MAPKs and inducing the expression of SIRT3 and SIRT4, rhamnetin provides protective effects. These properties suggest that rhamnetin may have therapeutic potential to protect the heart from ischemia-related injury [55].

A study published in 2020 suggests that DDUG (4,4′-diacetyldiphenylurea bis(guanylhydrazone)), a potent Chk2 inhibitor, inhibits the intracellular growth of *Mycobacterium tuberculosis*. However, the experimental data do not definitively determine whether the investigated substance acts through a Chk2-dependent mechanism or independently. Further exploration of other Chk2 inhibitors and investigation of the Chk2-CDC25A pathway may help resolve this issue [56]. Despite not being a substance of natural origin, the properties of DDUG indicate an interesting direction of research on checkpoint kinase 2 inhibitors, including plant-derived compounds.

Moreover, the recently published results of a study conducted on mice suggest that Chk2 inhibitors, such as DDUG, may have significant applications in the treatment of chronic inflammatory diseases caused by an excessive immune response. The findings of this study provide an interesting insight into the role of DNA damage response in the innate immune response. The study showed that the inhibition of Chk2 activity significantly alleviated chronic lung fibrosis resulting from acute organ damage induced by exposure to LPS [57]. This potential application of Chk2 inhibitors in chronic inflammatory diseases opens up new avenues for research and treatment.

### 4.1. Challenges and Limitations

Despite their therapeutic potential, plant-derived Chk2 inhibitors face many challenges and limitations, often concerning their status natural substances. Substances belonging to the flavonoid group have limitations due to several fundamental issues, such as chemical instability, low solubility, bioavailability, and specific pharmacokinetics influenced by liver metabolism and intestinal microflora. In addition, we have to face limitations resulting from plant production, such as very low production efficiency, difficulties in improving biosynthesis, and complicated isolation, extraction, and purification [58]. The fundamental problem is that flavonoids usually have limited oral bioavailability. This is primarily due to their poor solubility, insufficient absorption after oral administration, and intensive hepatic metabolism mediated by phase I and II enzymes. The above factors significantly reduce the chance achieving effective concentrations in vivo [59].

Moreover, despite the promising results of preclinical studies on the effects of Chk2 inhibitors, it is unknown how their effectiveness will translate into clinical practice. A significant problem will also be identifying and selecting patients for therapy and optimal dosage regimens [44,60].

### 4.2. Potential Future Developments

A compelling area to be investigated is the impact of DNA Damage Repair proteins (including Chk2 inhibitors) on the immune system. The inhibition of DDR proteins has the potential to produce a variety of context-dependent effects [45]. Using Chk2 inhibitors as immunomodulators in inflammatory diseases seems particularly intriguing and may provide new methods of treating these chronic diseases [57].

In the context of M. tuberculosis’s increasing resistance to antibiotic therapy, the interesting properties of the Chk2 inhibitor DDUG could offer a powerful weapon in the fight against tuberculosis [56].

However, checkpoint kinase 2 inhibitors seem to have the greatest potential in anticancer therapy. Enzymes and protein kinases involved in DDR are not just appealing targets for anticancer drugs, but also hold the potential to significantly improve the treatment of cancer diseases in the long term, providing a ray of hope in the fight against cancer [17,45,46,47].

Plant-derived Chk2 inhibitors, as shown by the studies mentioned above, are a legitimate alternative to other substances belonging to this group [19]. Unfortunately, due to the small amount of literature data, it is not easy to indicate the direction in which the development of these chemical compounds will go.

### 4.3. Key Findings and Directions for Further Research

Research into plant-derived Chk2 inhibitors has yielded promising results. Rutin and rhamnazin have shown potent inhibitory activity against Chk2 kinase [19]. Moreover, studies have highlighted the synergistic therapeutic effect of substances we can classify as plant-derived Chk2 inhibitors with conventional chemotherapeutic agents, suggesting their potential in combination therapy (Table 2). Combining rutin with other chemotherapy drugs may reduce the drug resistance of cancer cells and limit the side effects of the treatment. Additionally, rutin has been shown to inhibit the proliferation of many cancers, such as lung, breast, colon, and prostate. It should also be noted that rhamnazin enhances the chemotherapeutic action of sorafenib; however, this does not occur through Chk2 inhibition. An important issue to emphasize is the multifaceted nature of the action of natural compounds, so even though these substances exhibit Chk2 inhibitor properties in laboratory studies, their action on cancer cells may occur through entirely different pathways than Chk2 inhibition [19,43,61].

In summary, Chk2 inhibitors hold great promise as novel therapeutic agents for cancer therapy. Future research should address the challenges and limitations associated with their use, such as toxicity, specificity, and standardization, by optimizing chemical structures and integrating multidisciplinary approaches. Translational studies evaluating the efficacy and safety of Chk2 inhibitors in preclinical and clinical settings are warranted to validate their therapeutic potential and advance their clinical development. By harnessing the therapeutic potential of Chk2 inhibitors, we may pave the way for developing safer and more effective treatments for cancer and inflammatory diseases. An important issue is also the exploration of the therapeutic potential of substances of plant origin, which have a very complex effect on the human body based on many mechanisms. The complexity and number of interactions exhibited by substances belonging to flavonoids that have chk2 inhibitor activity are both an opportunity to develop modern pharmacological treatment methods and a challenge in research. However, it should be remembered that many drugs used in medicine have natural equivalents, and we should not forget the potential therapeutic possibilities of substances of natural origin.

## 5. Conclusions

Chk2 is an essential enzyme for regulating DNA repair and is often found in large quantities in tumor cells, contributing to their survival. By utilizing natural substances, researchers have identified potential inhibitors of Chk2, offering not only a possible anticancer therapy but a neuroprotective therapy for numerous neurodegenerative diseases. Moreover, natural compounds are beneficial, as they provide minimal potential side effects compared to current methods, such as chemotherapy or radiotherapy.

Experimental studies carried out in vitro and in vivo suggest a significant number of beneficial properties towards neurodegenerative diseases such as Alzheimer’s disease, Parkinson’s disease, and Huntington’s disease. By targeting Chk2, neurodegeneration is prevented, lost functions are restored, and neuron survival is improved. All in all, the inhibition of Chk2 may delay the onset of the disease, extend survival, and potentially enhance the quality of life of the people affected by neurodegeneration. Regarding cancer treatment, Chk2 inhibitors exert promising results, such as sensitizing tumor cells to the cytotoxic effects of chemotherapy and radiotherapy or attenuating hematological cytotoxicity induced by those therapies. On the other hand, Chk2 inhibitors desensitize normal cells to DNA damage caused by chemotherapy, which may indicate that there would be fewer side effects than conventional anticancer therapy.

However, further research is necessary to fully clarify the potential of the Chk2 inhibitors, including their efficacy and safety. There are too few research papers to decide whether or not natural compounds are, indeed, sufficient in neuroprotection and cancer therapy. Moreover, it should be mentioned that apart from the benefits, there may also be several limitations associated with the use of natural substances, such as the following:

- The lack of data indicating the optimal and toxic doses;
- The lack of data regarding their potential side effects;
- The lack of data evaluating their pharmacodynamic and pharmacokinetic properties.

Nonetheless, the inhibition of Chk2 kinase presents a promising strategy that could complement existing therapeutic approaches and enhance outcomes for patients with neurodegenerative and cancer diseases.

## Figures and Tables

**Table 1:** Mechanisms of inhibition of Chk2 by plant-derived substances.

Substance	Origin	Mechanism of Inhibition	Reference
**Flavonoids**			
Artemetin	*Cordia verbenacea*, *Cladanthus arabicus*	direct, by interlinkage with catalytic amino acid residues	[19]
Rhamnetin	*Camellia sinensis*, *Ammannia multiflora*	direct, by interlinkage with catalytic amino acid residues	[19]
Pachypodolol	*Melicope triphylla*, *Melicope semecarpifolia*	direct, by interlinkage with catalytic amino acid residues	[19]
Rhamnazin	*Callicarpa kwangtungensis*, *Halocnemum strobilaceum*	direct, by interlinkage with catalytic amino acid residues	[19]
Rutin	*Camellia sinensis*, *Amaranthus hybridus*	direct, by interlinkage with catalytic amino acid residues	[19]
**Polyphenols**			
Curcumin	*Curcuma longa*	indirect, by decreasing the expression of Chk2 mRNA	[20]
Resveratrol	*Humulus lupulus*, *Malus*	direct, by decreasing phosphorylation of Chk2	[21]
**Lactones**			
Xanthatin	*Xanthium pungens*, *Xanthium strumarium*	indirect, by downregulating the expression of Chk2	[22]

**Table 2:** Summary of biological properties discussed in this section.

Substance/Group of Substances	Biological Properties	Reference
Artemetin	Decreases cancer cell growth, induces apoptosis, inhibits cell migration, modulates microtubule dynamics, affects proteins like filamins, and leads to cytoskeleton disassembly.	[48]
Rhamnetin	Radiosensitization in NSCLC, inhibition of EMT, chemosensitization in HCC, anti-inflammatory and antioxidative effects.	[52,53,54,55]
Pachypodolol	Anti-inflammatory, antioxidant, anti-mutagenic, antimicrobial, antidepressant, anticancer, antiemetic, antiviral, cytotoxic.	[49]
Rhamnazin	Inhibits angiogenesis by targeting VEGFR2 signaling pathways.	[51]
Rutin	Anticarcinogenic, neuroprotective, antiproliferative, anti-inflammatory, antimetastatic, and antioxidative effects.	[43]
DDUG (4,4′-diacetyldiphenylurea bis(guanylhydrazone))	Inhibits intracellular growth of *Mycobacterium tuberculosis.*	[56]
Chk2 inhibitors	Sensitize tumor cells to DNA-damaging agents, induce cell cycle arrest and apoptosis, enhance efficacy of conventional cancer treatments, overcome drug resistance mechanisms, and have chemoprotective and radioprotective effects in non-tumor cells.	[17,45,46,47]
